# Systematics and Phylogenetic Placement of *Panicum* L. Species within the Melinidinae Based on Morphological, Anatomical, and Molecular Data (Poaceae, Panicoideae, Paniceae)

**DOI:** 10.3390/plants12020399

**Published:** 2023-01-14

**Authors:** Carolina Delfini, Diego L. Salariato, Sandra S. Aliscioni, Fernando O. Zuloaga

**Affiliations:** 1 Instituto de Botánica Darwinion (ANCEFN–CONICET), Labardén 200, Casilla de Correo 22, San Isidro B1642HYD, Argentina; cdelfini@darwin.edu.ar (C.D.); dsalariato@darwin.edu.ar (D.L.S.); saliscioni@darwin.edu.ar (S.S.A.); 2 Cátedra de Botánica General, Facultad de Agronomía, Universidad de Buenos Aires, Av. San Martín 4453, Buenos Aires C1417DSE, Argentina

**Keywords:** Africa, *Batochloa*, *Megathyrsus*, *Panicum deustum*, *Panicum trichocladum*, *Panicum vollesenii*

## Abstract

Generic boundaries of the African species *Panicum deustum* Thunb., *Panicum trichocladum* Hack. ex K. Schum., and *Panicum vollesenii* Renvoize are analyzed and compared with related genera of the tribe Paniceae and the subtribe Melinidinae. Based on morphological (vegetative and reproductive characters including habit, ligules, inflorescence, spikelets, and ornamentation of the upper anthecium), anatomical (transverse section of leaves), and molecular data (three chloroplast markers), a new genus is proposed for *P. deustum*, while *P. trichocladum* and *P. vollesenii* are transferred to the genus *Megathyrsus* (Pilg.) B.K. Simon & S.W.L. Jacobs. The phylogenetic position of both taxa within the Melinidinae and their morphological affinities with other genera of the subtribe are also discussed. Additional studies on the Melinidinae will clarify the systematic position of the genera that are still in a doubtful position within the subtribe, such as *Eriochloa* and *Urochloa.*

## 1. Introduction

Within the grass family Poaceae, the Paniceae, with the ages of the stem and the crown estimated to be in the Eocene and Oligocene, respectively, has an Afrotropical ancestral distribution and is dispersed and diversified pantropically since the late Oligocene [1]. Members of the Paniceae share a bi-flowered spikelet, their lower floret is male or neuter, and the upper floret is typically perfect and usually indurated; additionally, the taxa of this tribe mostly have a basic chromosome number of *x* = 9. This tribe includes five subtribes. Among them, the subtribe Melinidinae [2] has a total of 14 genera and three “incertae sedis” species of *Panicum* grex Deustum [3]. Of these, *Megathyrsus* (Pilg.) B.K. Simon & S.W.L. Jacobs is a genus with species in Africa and Eurasia [4], and *M. maximus* (Jacq.) B.K. Simon & S.W.L. Jacobs is an introduced species all over the world. *Tricholaena* Schrad., with two species, also has a distribution in Africa and Asia, and *Moorochloa* Veldkamp includes three species in Africa and Eurasia. Finally, *Melinis* P. Beauv., with nearly 23 species [4], has the conspicuously introduced species, *M. minutiflora* P. Beauv. and *M. repens* (Willd) Zizka, all over the world. *Thuarea* Pers. grows in Madagascar, Eurasia, and Australasia, while the monotypic *Eccoptocarpha* Launert and *Yvesia* A. Camus are present in Africa, the latter being restricted to Madagascar. Three genera are restricted to America: *Scutachne* Hitchc. & Chase is a monotypic genus endemic of Cuba, *Rupichloa* Salariato & Morrone comprehends two species that are restricted to Brazil, and *Chaetium* Nees has three species distributed from Mexico and the Caribbean to northern Brazil. Finally, two genera are cosmopolitan: *Eriochloa* Kunth, with approximately 33 species, and *Urochloa* P. Beauv., with more than 100 species, are in need of a taxonomic revision.

Early diversification of Melinidinae was placed through the early Miocene in the Afrotropics [1], while its monophyly has been recovered in several contributions works [1,5,6,7,8,9,10,11,12]. The genera of the subtribe have a basic chromosome number of *x = 9*, or less frequently a chromosome number of *x* = 8, and are C_4_ of the PCK subtype [13,14,15,16]: the PCK subtype is characterized anatomically by the presence of two vascular bundle sheaths, the outer parenchymatous bundle with specialized chloroplasts that are centrifugally disposed [17,18]. On the other hand, there is a significant variation in the morphology present in different genera of the subtribe. [11] reported two major clades within the Melinidinae: clade I includes *Rupichloa*, *Eriochloa distachya* Kunth, *Moorochloa*, *Tricholaena*, *Melinis*, *Leucophys*, and *P. deustum* Thunb., which are all taxa with a deciduous, cartaceous, or smooth upper anthecium, and clade II includes species of the paraphyletic genera *Urochloa* and *Scutachne*, and the polyphyletic genera *Eriochloa*, *Megathyrsus,* and *Chaetium*.

At the end of the last century, *Panicum* was a genus that still included a wide array of species [19,20], encompassing a wide variety in terms of exomorphological characters, anatomical characters, and basic chromosome numbers. In recent years, phylogenetic studies, on the basis of morphological and molecular data [5,6,7,20,21,22,23], have confirmed that *Panicum* is a polyphyletic genus; as a result, subgenera were raised to the generic level or new genera were established in different subtribes of the Paniceae (a summary can be seen in [20]).

Since species of *Panicum*, all using the C_4_ NAD–me photosynthetic subtype [20], were restricted to the subtribe Panicinae, there is a need for reevaluation of three species of the genus that remains within the subtribe Melinidinae: *P. deustum*, *P. trichocladum* Hack. ex K. Schum, and *P. vollesenii* Renvoize. We here aimed to define the systematic position, with the Melinidineae, of the three “incertae sedis” species of “*Panicum*”, based on new molecular evidence from the *ndh*F (NADH dehydrogenase subunit F), *rpl*16, and *trn*L-F genes, together with new morphological and anatomical data. The results supported the establishment of a new genus for *P. deustum* and the transfer of the remaining two species to the genus *Megathyrsus*. This study further clarified the systematics of a relevant group of grasses of economic importance in the world, with a phylogenetic study that solved the taxonomic position of several taxa in the subtribe.

## 2. Results

### 2.1. Phylogenetic Reconstruction

**Figure 1:**
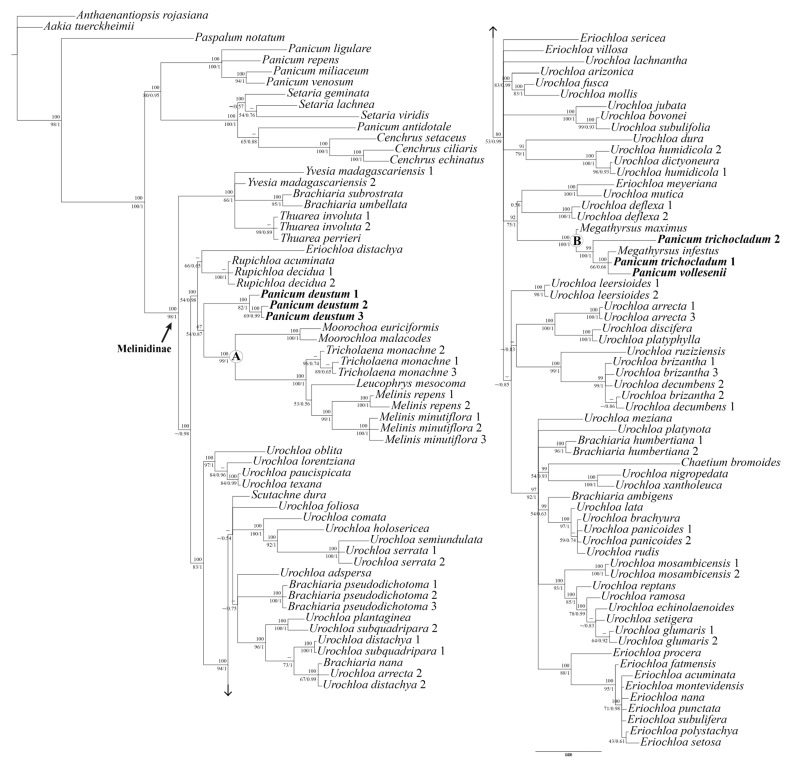
A 50% majority-rule consensus tree from the Bayesian inference analysis of the total combined dataset (*ndh*F + *rpl*16 + *trn*L-F). Bootstrap supports from parsimony are listed above the branches and bootstrap supports from maximum likelihood/posterior probabilities with Bayesian inference are listed below the branches. Nodes with “–” have bootstrap supports ˂ 50%. Clades denoted by letters are discussed in the text.

The general features and descriptive statistics of the DNA datasets used in the phylogenetic analyses are presented in Table S1 [available in supplementary material here]. Plastid *ndh*F region has the most variable dataset with 297 (13.94%) phylogenetically informative sites, followed by *trn*L-F and *rpl*16 which have 127 (10.83%) and 115 (9.68%) phylogenetically informative characters, respectively. The ILD test of the tree cpDNA combined does not indicate incongruence between partitions (ILD: P = 0.73). The strict consensus tree from MP, the 50% majority-rule consensus tree from BI, and the ML tree recovered using similar topologies show the same strongly supported clades; therefore, only the BI tree of the combined three-region DNA dataset is presented here (Figure 1). All aligned data matrices and trees of separate and combined datasets from the three methods of analysis are available at Repositorio Institucional CONICET Digital under the following link: http://hdl.handle.net/11336/17622, accessed on 23 December 2022.

We were able to include three accessions of *P. deustum*, which have identical or nearly identical sequences and are placed together by the three analyses. In the case of *P. trichocladum*, mutations in the sequences lead the two accessions to distinct placements, but both are included in *Megathyrsus*. We were able to amplify only a single voucher specimen of *P. vollesenii* that corresponds to the type collection of the name (Figure 1; Table S2 available in Supplementary Material here).

The topology of the three-region DNA dataset groups all the Melinidinae accessions analyzed in a strongly supported clade (Bayesian posterior probability (BPP) 1/ML bootstrap (MLB) 98/parsimony bootstrap (PB) 100), with *P. deustum*, *P. trichocladum* and *P. vollesenii* included in it (Figure 1). The backbone of the tree is not fully resolved, but all combined analyses placed *P. deustum*, *P. trichocladum,* and *P. vollesenii* into two clades, as detailed next.

The three accessions of *P. deustum* are clearly monophyletic (BPP 1/MLB 82/PB 100) and are moderately (BI) supported as a sister group to clade A (BPP 0.87/MLB 54/PB 67), which includes species of *Moorochloa*, *Tricholaena*, *Leucophrys* Rendle, and *Melinis*.

Clade B (“Megathyrsus clade”) is strongly supported and includes two species of *Megathyrsus*, *M. infestus* (Andersson) B.K. Simon & S.W.L. Jacobs and *M. maximus* (Jacq.) B.K. Simon & S.W.L. Jacobs), plus *P. vollesenii* and the two accessions of *P. trichocladum*. Within this clade, *Megathyrsus infestus*, *P. vollesenii,* and *P. trichocladum* 1 are placed in a polytomy (BPP 0.66/MLB 66/PB 100), with *P. trichocladum* 2 and *Megathyrsus maximus being* strongly supported as its successive sisters (BPP 1/MLB 100/PB 99; BPP 1/MLB 100/PB 100, respectively).

*Urochoa* and *Eriochloa* were resolved polyphyletically, with most species distributed in different clades throughout the tree and some in an isolated/uncertain position. The six unplaced “*Brachiaria*” species analyzed [i.e., *Brachiaria ambigens* Chiov., *B. humbertiana* A. Camus, *B. nana* Stapf, *B. pseudodichotoma* Bosser, *B. subrostrata* A. Camus, and *B. umbellata* (Trin.) Clayton] also do not form a clade, being in an isolated position or related to other Melinidinae taxa.

### 2.2. Anatomy and Morphology

#### 2.2.1. Foliar Anatomy of *Panicum deustum* (*Bequaert 3398, de Witte 10792*) 

**Figure 2:**
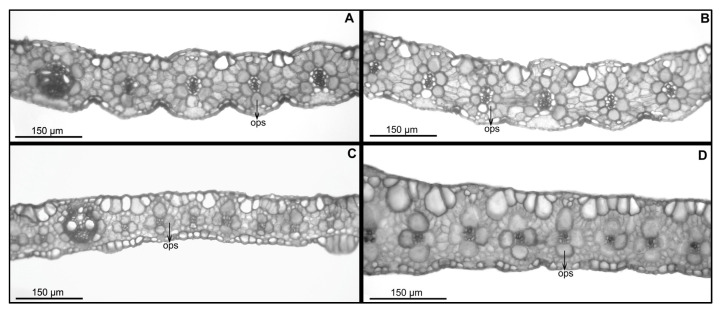
Light micrographs of leaf blade in cross section: (**A**,**B**). *Panicum deustum*; (**C**). *Panicum trichocladum*; (**D**). *Panicum vollesenii*. Abbreviations: ops = outer parenchymatous bundle [vouchers: (**A**): *Bequaert 3398*, (**B**): *de Witte 10792*, (**C**): *Hitchcock 24927*, (**D**): *Vollesen 3871*].

Transverse section (Figure 2A,B): The leaf outline is flat to very open V, with 90–160 μm in width and with ribs and furrows that are scarcely distinctive. The semicircular median keel developed with adaxial colorless parenchyma and some primary vascular bundles, generally three, and several second- and third-order vascular bundles; all bundles are in an abaxial position and are indistinguishable structurally from the lateral vascular bundles. The mesophyll with chlorenchyma cells conspicuously radiates in a single layer of cells around the vascular bundles; there are usually 2–3 mesophyll cells between contiguous vascular bundles. The vascular bundles have an outer parenchymatous sheath with globose cells and specialized chloroplasts in a centrifugal position, complete or interrupted by abaxial girds of sclerenchyma in the first-order vascular bundles. There are generally parenchymatous bundle cells of second- and third-order vascular bundles that are similar in size to first-order vascular bundles, with 9–12 parenchyma sheath cells surrounding first-order vascular bundles and 6–8 cells surrounding second- and third-order vascular bundles. The inner mestome sheath has cell walls that are thickened and has no chloroplasts in all vascular bundles. Very small strands of adaxial and/or abaxial sclerenchyma are associated with minor vascular bundles. Bulliform cells form well-defined and regular groups of 2–6 cells between consecutive vascular bundles or above third-order vascular bundles, occupying approximately ¼ of the leaf thickness.

#### 2.2.2. Foliar Anatomy of *Panicum trichocladum* (*Eichinger 5876*, *Hitchcock 24927*) and *P. vollesenii* (*Vollesen 3871*) 

Transverse section (Figure 2C–D): The leaf outline is flat to very open V, with 70–120 μm in width and with ribs and furrows that are scarcely distinctive. The semicircular median keel developed with scarce adaxial colorless parenchyma and one first-order vascular bundle in *P. vollesenii* or abundant adaxial colorless parenchyma and three first-order vascular bundles in *P. trichocladum*. In both species, there are some minor-order vascular bundles. All bundles are in an abaxial position, indistinguishable structurally from the lateral vascular bundles. The mesophyll with chlorenchyma cells radiates or conspicuously radiates in a single layer of cells around the vascular bundles; there are generally 1(–2) mesophyll cells between contiguous vascular bundles. The vascular bundles have outer parenchymatous sheath with globose cells and specialized chloroplasts in a centrifugal position, complete or abaxially interrupted by the girds of sclerenchyma in the first-order vascular bundles. There are parenchymatous bundle cells of minor vascular bundles that are bigger than first-order vascular bundles, with 8–10 parenchyma sheath cells surrounding the first-order vascular bundles and commonly 4(–6) cells surrounding the minor-order vascular bundles; these cells are arranged in a cross and, with the adaxial and abaxial cells, are somewhat winged. The inner mestome sheath has cell walls that are thickened and has no chloroplasts in all vascular bundles; in some cases, there is no differentiation between second- and third-order vascular bundles. Very small strands of adaxial and/or abaxial sclerenchyma are associated with minor vascular bundles. Bulliform cells form well-defined and regular groups of 2–4 cells between consecutive vascular bundles, occupying approximately ¼ of the leaf thickness; there are adaxial and abaxial macrohairs with cushion based in *P. trichocladum*.

#### 2.2.3. Ornamentation of the Upper Anthecia

**Figure 3:**
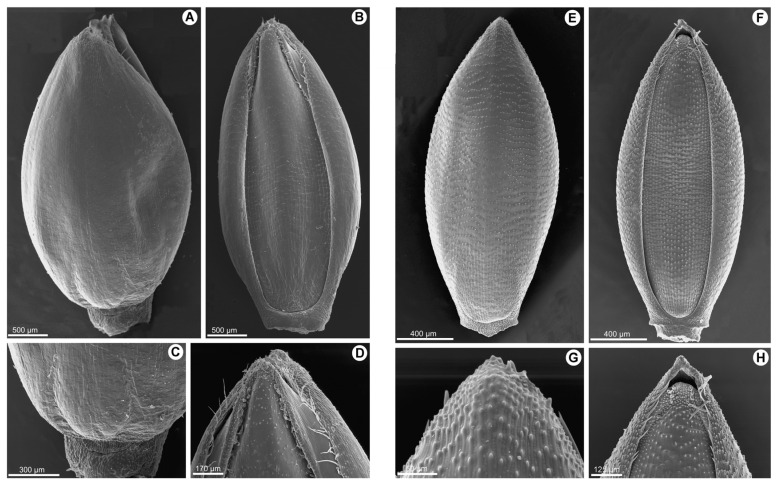
(**A–D**). Scanning electron micrographs of the upper anthecium of *Panicum deustum*: (**A**). dorsal view; (**B**). ventral view; (**C**). base of the upper lemma; and (**D**). apex of the upper palea. (**E–H**). Scanning electron micrographs of the upper anthecium of *Panicum trichocladum*: (**E**). dorsal view; (**F**). ventral view; (**G**). apex of the upper lemma; and (**H**). apex of the upper palea [vouchers: (**A**–**D**): *Bequaert 3398*; (**E**–**H**). *Hitchcock 24927*].

The upper lemma of *P. deustum* shows a smooth surface without longitudinal or transverse ridges (Figure 3A–D), with simple papillae of 2–4 μm associated with the transverse anticlinal wall zone (one on each wall). Alternatively, in *P. trichocladum* and *P. vollesenii*, the ornamentation pattern in the upper lemma is characterized by prominent transverse pustules (Figure 3E–H), while longitudinal pustules are absent or very slight. Generally, a single papilla of 5–8 μm is associated with the transverse anticlinal wall.

## 3. Discussion

The particular configuration of the outer bundle sheath associated with the third-order vascular bundles in *P. trichocladum* and *P. vollesenii* is a diagnostic character that confirms the transfer of both species to the genus *Megathyrsus*. Similar to all Melinidinae, both species of *Panicum, Megathyrus infestus*, and *M. maximus* are C_4_ of the PCK subtype [13,14,15,16] but differ by the presence of usually four cells that surround the minor vascular bundles; therefore, Kranz parenchyma cells are arranged in a cross, frequently with the adaxial and abaxial cells being somewhat expanded and the vascular tissue looking square in transection. Other species that potentially could be included in this group, at least regarding the foliar anatomy, are *Panicum chusqueoides* Hack. (*Brachiaria chusqueoides* (Hack.) W.D. Clayton and *Brachiaria grossa* Stapf ([18]; Aliscioni, pers. obs.).

On the contrary, *P. deustum* lacks these anatomical characters. In this species, there are usually 6–8 Kranz parenchyma cells that surround minor-order vascular bundles, i.e., adjusted to a more classic pattern of the C_4_–PCK subtype. The other genera that integrate the sister clade of *P. deustum* all have a smooth upper lemma and also share a similar foliar anatomy. Previously, [18] mentioned these anatomical similarities, particularly for *Moorochloa eruciformis*, *M. malacodes* (named as a species of *Brachiaria* in his work) and *Tricholaena*; a similar foliar anatomy is present in *Leucophrys mesocoma* [11] and *Melinis minutiflora* [24], the latter with bulliforms associated with colorless mesophyll cells [25].

*Panicum deustum* is characterized by its lanceolate, cordate to subcordate leaves and inflorescence terminal, which is lax and open, and is usually covered with clavellate hairs. The spikelets are long ellipsoid, with the lower glume being 5-nerved; the upper glume, and lower lemma are subequal, being 5–7-nerved. The lower palea is developed, the lower flower staminate, and the upper anthecium is smooth, shiny, and indurate, with macrohairs at the top of the upper lemma (Figure 3A–D). This species has a basic chromosome number of *x* = 9.

Within the Melinidinae, the genus *Chaetium*¸ including three American species, is defined by its spiciform inflorescences, with spikelets that are dorsiventrally compressed and with a callus at the base; the glumes are awned, the lower one is long and lanceolate to setaceous, and the upper anthecium papillose all over its surface. The genus *Eccoptocarpha*, with one African species, includes an annual plant. It has racemose inflorescences and few flowers; the spikelets are dorsiventrally compressed and adaxial; the lower glume is ¾ of the length of the spikelet; the upper glume and lower lemma have transverse veinlets; and the upper anthecium is stipitate, being shorter than the upper glume and the lower lemma, smooth, shiny, glabrous and indurate. The polyphyletic genus *Eriochloa*, cosmopolitan and with nearly 35 species [4] in need of a revision, comprehends species with racemose to open and lax inflorescences. The spikelets are dorsiventrally compressed; the lower glume is reduced to a globose callus at its base; and the upper anthecium is longitudinally striate and has verrucose papillae, is mucronate or aristulate, indurate, and pilose toward the apex or glabrous. The genus *Leucophrys*, with two African species, is defined by its racemose inflorescences; the spikelets are appressed and adaxial, and dorsiventrally compressed, the lower glume is nearly as long as the upper glume, being 1–5-nerved; the upper glume and lower lemma are pilose; and the upper anthecium is smooth, shiny, glabrous, and indurate. *Megathyrsus* is an African genus, with ca. three species, related to *Urochloa*, and is characterized by its open and radiated inflorescences; the spikelets are dorsiventrally compressed and not stipitate; the lower glume is ⅓ to ¼ of the length of the spikelet, being (1–)3-nerved; and the upper anthecium is transversely rugose, crustaceous, mucronate, and glabrous. The genus *Melinis*, with approximately 30 species distributed in Africa and Asia, is introduced in America and includes species with lax and open inflorescences; the spikelets are laterally compressed and articulated below the upper anthecium; the lower glume is less than ⅕ of the length of the spikelet and is nerveless; and the upper anthecium is smooth, shiny, muticous, glabrous, herbaceous to cartilagineous. The genus *Moorochloa*, with three species distributed in Africa and Asia, is introduced elsewhere and has spikelets that are arranged in unilateral racemes, dorsiventrally compressed, and articulated below the upper anthecium; the lower glume is less than ⅕ of the length of the spikelet, being 1-nerved; and the upper anthecium is smooth, shiny, muticous, glabrous, and cartilagineous. *Rupichloa* is a genus restricted to Brazil with two species, which is defined by its open and lax inflorescences; the spikelets are dorsally compressed and stipitate; the lower glume is ½ to ¾ of the length of the spikelet, being 5–7-nerved; and the upper anthecium is longitudinally striate, crustaceous, covered with verrucose papillae, crestate, and has flattened macrohairs toward the apex. *Scutachne*, which is monotypic and has one species restricted to Cuba, is distinguished by having spikelets that are dorsiventrally compressed and stipitate, the lower glume is ½ of the length of the spikelet and is 5–7-nerved, and the upper anthecium is longitudinally striate, papillose, with the apex crested and pilose. *Thuarea*, with two species from Madagascar, Australia, and Asia, includes andromonoic plants, has racemose inflorescences and is entirely deciduous at maturity; the rachis is winged and ending in a naked point; the spikelets are adaxial; the lower glume is absent or obscure and nerveless; and the upper anthecium is smooth, indurate, muticous and pilose at the apex. The genus *Tricholaena*, with four species from Africa, Madagascar, and Eurasia, has open and lax inflorescences, with spikelets that are laterally compressed; the lower glume is less than ⅕ of the length of the spikelet, being 0–1-nerved; and the upper anthecium is smooth, shiny, glabrous, and muticous. *Urochloa*, a cosmopolitan genus with nearly 100 species, differs by its radiated or racemose inflorescences, with spikelets that are dorsiventrally compressed and are stipitate or not; the lower glume is ⅙ to the same length of the spikelet, being (0–)3–7(9–11)-nerved; and the upper anthecium is transversely rugose or longitudinally striate, with simple or verrucose papillae, and is indurate, mucronate to crestate or aristulate. Finally, *Yvesia*, a monotypic genus endemic to Madagascar, includes annual plants with racemose and digitate inflorescences and few flowers; the spikelets are dorsiventrally compressed, falling entire at maturity; the lower glume is absent or vestigial and is nerveless; and the upper anthecium is smooth, indurate, glabrous, and mucronate.

The distinguished characters of *P. deustum*, summarized in the following key points, allowed us to conclude that the species represents a new genus within the Melinidinae (i.e., *Batochloa* Salariato & Zuloaga). Regarding *P. trichocladum* and *P. vollesenii*, both species share the following characters with *Megathyrsus*: inflorescences lax and open, with spikelets dorsiventrally compressed, long ellipsoid, lower glume reduced, ⅕ to ⅓ nerved, nerveless to 3-nerved, upper glume and lower lemma 5–7-nerved, and upper anthecium transversely rugose and indurate (Figure 3E–H). Therefore, both species are transferred to the genus *Megathyrsus*.

**Key points to the genera of Melinidinae:**
1. Lower and upper glume awned
***Chaetium***
1′. Lower and upper glume muticous22(1′). Plants andromonoic; inflorescences deciduous as a whole at maturity; rachis broadly winged, foliaceous, and folded transversely to form a capsule
***Thuarea***
2′. Plants not andromonoic; inflorescences not early deciduous; rachis triquetrous, subcylindric, or broadly winged33(2′). Upper anthecium smooth, shiny, and coriaceous to membranous or hyaline; apex of the upper anthecium muticous43′. Upper anthecium longitudinal or transversely rugose, with simple or verrucose papillae all over its surface, coriaceous to crustaceous; apex of the upper anthecium mucronate, crestate, or awned104(3). Spikelets adaxial54′. Spikelets abaxial65(4). Upper anthecium not stipitate, nearly as long as the upper glume and the lower lemma; upper glume and lower pilose, without transverse veinlets
***Leucophrys***
5′. Upper anthecium stipitate, shorter than the upper glume and lower lemma; upper glume and lower lemma glabrous, with transverse veinlets
***Eccoptocarpha***
6(4′). Plants annual; inflorescences digitate
***Yvesia***
6′. Plants perennial; inflorescences not digitate77(6′). Upper anthecium falling with glumes and lower anthecium, not early deciduous, and laterally compressed
***Melinis***
7′. Upper anthecium early deciduous, without the bracts of the spikelet, dorsiventrally compressed88(7′). Inflorescence racemose, not pyramidal, with spikelets secund on short pedicels
***Moorochloa***
8′. Inflorescences lax, pyramidal, with spikelets not secund on long, capilliform pedicels99(8′). Inflorescence without glandular hairs; lower glume absent or vestigial; upper anthecium readily deciduous
***Tricholaena***
9′. Inflorescence with glandular hairs; lower glume ⅘ to nearly as long as the spikelet; upper anthecium not readily deciduous
***Batochloa***
10(3′). Spikelets with a globular bead-like callus at its base
***Eriochloa***
10′. Spikelets without a globular bead-like callus at its base1111(10′). Upper anthecium longitudinally rugose, with flat macrohairs at the apex1211′. Upper anthecium transversely rugose to slightly or strongly reticulate, without flat macrohairs at the apex1312(11). Spikelets arranged in racemose inflorescences. Endemic to the Caribbean
***Scutachne***
12′. Spikelets dispersed in open, and no racemose inflorescences. Brazil
***Rupichloa***
13(11′) Inflorescences lax, pyramidal, with third-order branches present
***Megathyrsus***
13′. Inflorescences racemose or lax, but only with first- and second-order branches
***Urochloa***


### Taxonomic Treatment

***Batochloa*** Salariato & Zuloaga, **nov. gen.** Type species: ***Batochloa deusta*** (Thunb.) Salariato & Zuloaga.

**Figure 4:**
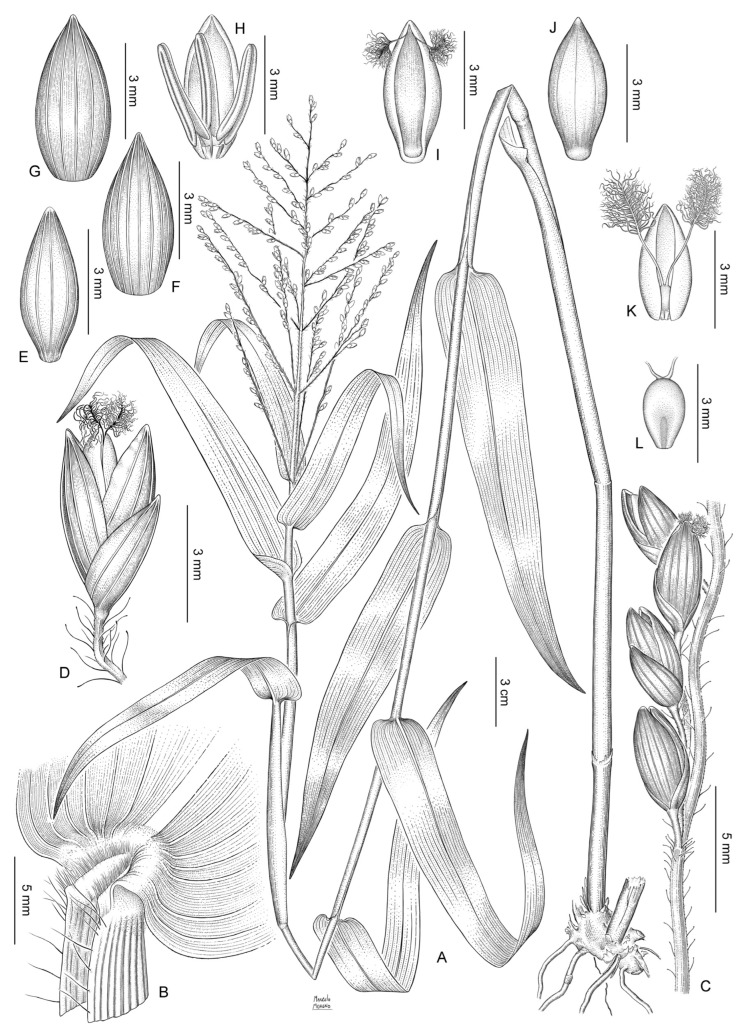
*Batochloa deusta* (Thunb.) Salariato & Zuloaga: (**A**). habit; (**B**). ligular region; (**C**). portion of the inflorescence with clavellate hairs; (**D**). spikelet, lateral view and pedicel with clavellate hairs; (**E**). lower glume, dorsal view; (**F**). upper glume, dorsal view; (**G**). lower lemma, dorsal view; (**H**). lower palea and lower flower with three stamens; (**I**). upper anthecium, ventral view; (**J**). upper anthecium, dorsal view; (**K**). upper palea with lodicules and gynoecium; and (**L**). caryopsis, hilum view [voucher: (**A**–**L**): *Bequaert3398*].

Shortly rhizomatous perennial with culms decumbent, rooting at the lower nodes. Ligules membranous. Blades lanceolate. Inflorescence a lax panicle with spikelets not secund arranged along first- and second-order branches, with glandular hairs on the main axis and branches. Spikelets long ellipsoid to ovoid. Lower glume as long as the spikelet, pilose, 5-nerved. Upper glume and lower lemma subequal, 5–7-nerved, the lower glume with long macrohairs toward the apex. Lower flower and lower palea absent. Upper anthecium ellipsoid, with bicellular microhairs at the apex of lemma, the whole surface covered by conspicuous papillae (Figure 3A–D; Figure 4).

*Etymology.* The name of the new genus honors Dr. Maria “Bat” Vorontsova, for her important contributions to the knowledge of grasses of Africa and Madagascar.

***Batochloa deusta*** (Thunb.) Salariato & Zuloaga, **nov. comb.** *Panicum deustum* Thunb. Prod. Pl. Cap.: 19. 1794. TYPE: South Africa, 1772–1775, *Thunberg s.n.* (holotype, UPS V-001844!; isotypes, LD1241117!, S-G-4485!, S-G-4486!). Figure 4.

*Panicum leptocaulon* Trin., Mém. Acad. Imp. Sci. Saint-Pétersbourg, Sér. 6, Sci. Math., Seconde Pt. Sci. Nat. 3, 1(2–3): 275. 1834. TYPE: East Africa. “V. sp. Afric. Oriental.” (holotype, LE!).

*Panicum unguiculatum* Trin., Mém. Acad. Imp. Sci. Saint-Pétersbourg, Sér. 6, Sci. Math., Seconde Pt. Sci. Nat. 3, 1(2–3): 275. 1834. TYPE: [South Africa. Cape]. “V. sp. Cap. b. sp.” (not seen).

*Panicum corymbiferum* Nees ex Steud., Syn. Pl. Glumac. 1: 76. 1853. TYPE: Mozambique. Delagoa Bay, 1822, *Forbes s.n.* (isotypes, K000282470!, K000282472!).

*Panicum arundinifolium* Schweinf., Bull. Herb. Boissier, sér. 2, 2: 22. 1894. TYPE: Eritrea. Ginda, Seitenthal, 1891, *G. Schweinfurth 435* (holotype, B10_1157139!).

*Panicum pubivaginatum* K. Schum., Pflanzenw. Ost-Afrikas: 102. 1895. TYPE: Tanzania. Usambara Mountains, July 1893, *Holst 8816* (lectotype, here designated, K000255544!; isolectotypes, M0103863!, W18940006548!). Other syntype: Zanzibar, *Hildebrandt 1187*.

*Panicum menyharthii* Hack., Bull. Herb. Boissier, sér. 2, 1: 766. 1901. TYPE: Mozambique. Boruma, July 1891, *L. Menyhart 902* (holotype, W19160024078!; isotypes, US00148294!, WU0042983!, WU0042984!, WU0042982!).

*Panicum deustum* var. *eburneum* Chiov., Annuario Reale Ist. Bot. Roma 8: 306. 1903. TYPE: Kenya. Kisumu-Londiani District: Fort Ternan, 20 January 1938, *P. Davoli 158* (holotype, FT000231!; isotype, FT000231!).

*Panicum deustum* var. *hirsutum* Peter, Repert. Spec. Nov. Regni Veg. Beih. 40 (1, Anhang): 42. 1929. TYPE: Tanzania. “Deutsch-Ost Afr. Kilimandscharo: Neu Moschi—RauWald...”, 3 July 1926, *A. Peter 42246* (holotype, B †; lectotype here designated, W19600020862!).

Perennial, caespitose, shortly rhizomatous. Culms 70–200 cm long, internodes cylindrical, hollow, 8–14 cm long, glabrous to shortly pilose near the nodes; nodes compressed, shortly pilose to glabrous. Sheaths striate, longer than the internodes, the margins with tubercullate hairs, shortly hispid at the upper portion; collar densely and shortly pilose. Ligule membranous-ciliate, 1.5–1.8 mm long, the membranous portion ca. 1 mm long. Blades lanceolate, 13–45 × 0.6–2.5 cm, subcordate to cordate, flat, acuminate, shortly hispid on both surfaces or the adaxial surface shortly hispid to glabrous, mid-nerve conspicuous, the margins ciliate toward the base, scabrous simple or broadly rounded, linear or lanceolate, 15–48 cm long, 5–35(–40) mm wide, glabrous or pilose, acuminate. Inflorescence terminal, a lax panicle with spikelets not secund, arranged in racemes along first- and second-order branches, 8–38 × 5–25 cm; peduncle 10–30 cm long, terete, with long clavellate hairs, occasionally glabrous; first-order branches subopposite to alternate, ascending or spreading, covered with clavellate hairs; spikelets solitary or in pairs in second-order scaberulous branches with clavellate hairs; pedicels slender, 1–7 mm long. Spikelets long ellipsoid to ovoid, dorsally compressed, obtuse, 3.8–4.8(–5.4) × 1.2–1.5 mm, glabrous, greenish; lower glume 3–4.5 mm long, more than ½ the length of the spikelet, embracing the upper glume, 5–7-nerved, acute; upper glume and lower lemma subequal, upper glume 7–9-nerved, acute; lower lemma glumiform, 5–7-nerved; lower palea elliptic, as long as the lower lemma, hyaline, glabrous; lower flower male, anthers 3, 2–4 mm long; upper anthecium ellipsoid, 3.6–4.7 × 1.2–1.7 mm, smooth, pale, indurate, with macrohairs and short prickles at the apex.

*Distribution and ecology.* Africa (Ethiopia to South Africa), Temperate Asia, and South America (introduced).

*Observations.* This species has a chromosome number of 2*n* = 36 [26]. The specimen *Holst 8816*, from K, is designated as a lectotype of *P. pubivaginatum* K. Schum., among the two syntypes originally cited in this species, since it is a full specimen and agrees with the protologue.

The specimen *Peter 42246*, from W, is designated as a lectotype of *P. deustum* var. *hirsutum* since this specimen fully agrees with the protologue and the original collection at B was destroyed during WW2. This species has peculiar glandular, clavellate hairs on the main axis, and branches of the inflorescence, which is a character also present in the genus *Adenochloa* Zuloaga, with 14 species distributed in Africa and Madagascar, and differing from the genus *Batochloa* by including non-Kranz species.

*Additional material examined.* BURUNDI. Kininya Mosso, 17 June 1952, *Michel 2897* (BR). DEMOCRATIC REPUBLIC OF THE CONGO. Kabenyi, *Bredo 1796* (BR, P); Beni, 1919, *Bequaert 3398* (BR); Kigoma lieu lit près Balozi, 15 July 1954, *de Witte 10792* (BR). ETHIOPIA. Gamu-Gofa Region, Mago National Park, ca. 5 km NE of the park headquarter along a trail towards Kinka, Combretum woodland on sloping ground, soil gravelly, derived from granite rocks, 19 December 1998, *Friis 9510* (K); Prov. Harar. Oromiya, west of Daletii, 15 November 1963, *Burger 3371* (US). KENYA. Nairobi to Nanyuki, moist forest, 10 September 1929, *Hitchcock 24749* (US); Nairobi National Park, shade near the hippo pool, 5 May 1959, *McCallum Webster K65* (US); Distr. Samburu, Mount Nyiru Mt. Nyiru, Ri-parat Forest Zone, 1 April 1995, *Bytevier 302* (BR). MOZAMBIQUE. Ad Sinum Delagoa, 1822, *Forbes s.n.* (BR); Manica, entre Dombe e Sam[banke], a 11 km do Dombe, margens do R. Mucombe ao Matindire, 21 Oct 1953, *Gomes Pedro 4515* (BR). RWANDA. Montagnes a l’est de Kisenyi, Nyondo, *Humbert 8444bis* (P); Pref. Kigali. Bugesera, environs de Karama, sur la piste de Karama à la route de Kigali, 12 March 1972, *Bouxin 1366* (BR). SENEGAL. Without locality, *Leprieur s.n.* (P 02251162). SOUTH AFRICA. Cape. Kaaimans river, *Wilman s.n.* (P 02726832); 6 miles northwest of Grahamstown, 8 December 1952, *Godfrey & Story SH-1353* (US); banks of the Sunday River, Graaft Reinet Div., 1840, *Drège s.n.* (K); Oribi Gorge Nature Preserve, 10 October 1963, *Swallen 10584* (US). TANZANIA. Mbulu District, *Hukui 63* (K, P); Kijuju estate, Kilimanjaro, 4 April 2002, *Hemp 3444* (UBT); Arusha; Ngorongoro Conservation Area, rim of Ngorongoro Crater, 19 June 2012, *Peterson* et al. *24311* (US); Moshi, 1 September 1929, *Hitchcock 24550* (US). UGANDA. Mbarasa, Ruisi falls, on edge of river, September 1925, *Maitland 768* (K, US); Jinja, edge of forest, 18 Sep 1929, *Hitchcock 24955* (US); Kaward Hill, near Kampala, November 1937, *Chandler 1993* (BR). ZAMBIA. Chilanga distr. Quien lake, in the irrigation wood in shade, 3 Oct 1929, *Sandwith 25* (K, US). ZIMBABWE. Victoria, Masvingo, forested hills just above Glenglivet Hotel, along path in secondary forest, 15 February 1974, *Davidse 6653* (MO, US); Melsetter. South facing road cutting E of Umvumvumvu bridge in Mutambara Tribal Trust Lands, 6 April 1969, *Crook 864* (BR).

***Megathyrsus trichocladum*** (Hack. ex K. Schum.) Salariato & Zuloaga, **nov. comb.** *Panicum trichocladum* Hack. ex K. Schum., Pflanzenw. Ost-Afrikas 5c: 103. 1895. TYPE: Tanzania. Kilimanjaro, Grasflächen oberhalb des Urwaldes, July 1887, *H. Meyer 140* (lectotype here designated, B10_0715462!; isolectotype, US00140067!, fragment ex B). Other syntypes: Tanzania. Usambara, 1893, *Volkens 69* (BR0000008766779!, US00140067!, fragment ex B).

*Panicum simbense* Mez, Bot. Jahrb. Syst. 57: 186. 1921. TYPE: Africa. Tanzania. Usambara Mountains, *Holst 378* (lectotype here designated, US00139998!, fragment ex B). Other syntype: Tanzania. Usambara Mountains, *F.L. Stuhlmann 28* (US00139998!, fragment ex B).

*Panicum protractum* Peter, Repert. Spec. Nov. Regni Veg. Beih. 40 (1, Anhang). 42, Table S2, Figure 1. 1929, nom. illeg. hom., non Mez, 1917. TYPE: “Deutsch Öst. Afr., Kili-mandscharo: Rau-Fl [to] Alt Moschi...” *Peter 42203* (not located, presumably destroyed at B).

*Panicum trichocladum* var. *parviflorum* Peter, Repert. Spec. Nov. Regni Veg. Beih. 40 (1, Anhang): 191. 1938. TYPE: “Deutsch-Ostafrika: Winterhochland; im Kraterkessel Ngorongoro—Laroda, im Wasser...”, *A. Peter 43179* (not located).

Plants perennial, shortly rhizomatous, with culms that are decumbent and rooting at the lower nodes, 30–240 cm long, branching at the lower and upper nodes; internodes 4– 10 cm long, hollow, terete, glabrous, and rigid; nodes dark, compressed, glabrous. Sheaths striate, 3–14 cm long, longer or shorter than the internodes, covered with tuber-cullate hairs to glabrous; collar shortly pilose. Ligules membranous-ciliate, 1–2 mm long. Blades lanceolate, 4–10(–17) × 0.3–0.6(–1.3) cm, flat, subcordate, acuminate, pilose to gla-brous on both surfaces, the margins scabrous, the lower ones ciliate near the ligular region, midnerve manifest. Inflorescence terminal; peduncle up to 20 cm long, cylindrical, gla-brous or with long hairs near the panicle; panicle lax, open, 8–10 × 5–7 cm; axis of the inflorescence cylindrical, covered, or sparse, with long whitish tubercullate hairs; first-order branches alternate, divergent, axis of the branches with long tubercullate hairs, oc-casionally sparse, spikelets solitary or paired on claviform and pilose pedicels, 1–7 mm long. Spikelets long ellipsoid, dorsally compressed, (2.2–)2.6–2.8 × 1–1.1 mm, falling en-tire, pale to greenish, glabrous; lower glume 0.2–0.5 mm long, less than ⅕ of the length of the spikelet, nerveless, truncate or obtuse; upper glume and lower lemma similar, upper glume 5-nerved, acute, lower lemma glumiform, 5–7-nerved; lower palea as long as the lower lemma, hyaline, glabrous; lower flower male, anthers 3, 1.8 mm long; upper anthe-cium ellipsoid, 2.2–2.6 × 1–1.1 mm, indurate, pale, transversely rugose; upper lemmawith involute margins over the upper palea.

*Distribution and ecology*. Africa, present from Ethiopia and Sudan to Democratic Republic of the Congo, Kenya, Tanzania, Uganda, Zambia, Zimbabwe, Malawi, and Mozambique; it is introduced in Tropical Asia and South America. It grows in bush or forest shade, and on stony or sandy soils.

*Observations.* This species has a chromosome number of 16 [27]. *Panicum trichocladum* was described on the basis of two syntypes, Meyer 140 and *Volkens 69*, both from Tanzania. Of these, *Meyer 140* from B (B 10 0715462), which is a full specimen in agreement with the protologue, is designated here as a lectotype of the species.

*Panicum simbense* was described on the basis of two syntypes, *Stuhlmann 28*, and *Holst 378*. Both specimens are not extant at B, presumably lost during WW2. Therefore, a lectotype of *Holst 378*, which agrees with the protologue, is designated from US, a fragment of the original material at B.

*Representative specimens examined.* BURUNDI. Bujumbura, 24 Mar 1975, *Lambinon 75/33* (BR); Bujumbura, jachères (Ndahangwa), 1 June 1967, *Lewalle 1994* (BR). COMORES. Réserve Forestière du Benara, 28 November 2000, *Labat* et al. *3302* (P). DEMOCRATIC REPUBLIC OF THE CONGO. Prov. Tshopo. Yangambi, *Blomme 74* (BR); Parc National de la Garamba, Nagero, 1 April 1952, *Troupin 646* (BR)¸ Makokoma, 17 August 1919, *Bequaert 5311* (BR). KENYA. Kakamega Forest, June 1961, *Lucas 119* (FT). MALAWI. Nyasaland, Ruo River, Chiromo, 28 November 1950, *Jackson 315* (K, US); Nkata Bay, 6 July 1952, *Jackson 914* (BR). RWANDA. Eastern Prov. Mutara, colline Cyabayaga, galerie pres de la riv. Kakitumba, 12 February 1958, *Troupin 6032* (BR). TANZANIA. Amani, *Eichinger 5876* (US); Mvumo Hill, mile 15 from Hq., 6 July 1973, *Greenway & Kanuri 15368* (MO); Pangani Distr.: Koka (Zigua). Kilimamgwido area, 27 August 1973, *Bond 121* (BR, US); Marangu, July 1893, *Volkens 584* (BR). UGANDA. Distr. Kampala. Kampala, 17 September 1929, *Hitchcock 24927* (US); Entebbe, 16 September 1929, *Hitchcock 27820* (US).

***Megathyrsus vollesenii*** (Renvoize) Salariato & Zuloaga, **nov. comb.** *Panicum vollesenii* Renvoize, Kew Bull. 35(1): 202. 1980. TYPE: Tanzania. Liwale Distr., ca. 10 km NNW of Kingupira, 125 m, 3 August 1976, *K. Vollesen 3871* (holotype, C10001154!; isotypes, EA (not seen), K000255557!, WAG0001529!).

 Plants perennial, shortly rhizomatous, with culms trailing or scrambling, 100–150 cm long, wiry, branched; internodes terete, hollow, glabrous. Sheaths striate, open. Ligule membranous-ciliate. Blades lanceolate, 4.5–9 cm long, 5–7 mm wide, cordate, flat, glau-cous, the margins scabrous, apex acuminate. Inflorescence an open and lax panicle, 9–14 cm long, and ovate; primary panicle branches spreading, panicle branches stiff, pilose; spikelets solitary on claviform pedicels 1–4 mm long, scaberulous. Spikelets long ellipsoid, dorsally compressed, 2.8–3 × 1 mm, glabrous, greenish, acute; lower glume 1 mm long, 3- nerved, acute; upper glume and lower lemma subequal, membranous; upper glume 1 mm long, ⅓ of the length of the spikelet, 1–3-nerved, membranous, acute; upper glume 5–7- nerved, acute; lower lemma glumiform, 5-nerved, acute; lower palea elliptic, 2.5 × 0–9 mm, hyaline, glabrous; lower flower male, anthers 3; upper anthecium ellipsoid, 2.5–2.7 × 0.9 mm, dorsally compressed, indurate, pale, transversely rugose over the lemma and palea; upper lemma 5-nerved, the margins involute; the upper palea involute, indurate. 

*Distribution and ecology.* Tanzania, only known from the type material collected at a riverine forest [28].

*Observation.* This species is morphologically related to *P. trichocladum*, the latter differing by its reduced, nerveless, and obtuse-to-truncate lower glume [28].

## 4. Materials and Methods

### 4.1. Phylogenetic Reconstruction

#### 4.1.1. Taxon Sampling

The DNA data matrix used here consisted of a total of 125 accessions, of which 111 are ingroup corresponding to the Melinidinae taxa. Previously published chloroplast DNA (cpDNA) *ndh*F, *rpl*16, and *trn*L-F matrices [11], excluding the outgroup, were completed with new sequences downloaded from GenBank (42, 5, and 4, respectively), plus the new accessions of *P. deustum*, *P. trichocladum* and *P. vollesenii* that were sequenced for this study [Table S2, available in Supplementary Material here]. In addition, 14 species belonging to six closely related genera were selected as the outgroup, based on [11,12,29]: *Aakia* J.R. Grande, *Anthaenantiopsis* Mez ex Pilg., *Cenchrus* L., *Panicum*, *Paspalum* L., and *Setaria* P. Beauv. The information about the vouchers and accession numbers of the new sequences obtained for this study and those downloaded from GenBank is presented in Table S2.

#### 4.1.2. DNA Amplification and Sequencing

Total genomic DNA was extracted from herbarium specimens using modified CTAB protocols from [30]. Some DNA extractions were conducted using the DNeasy Plant Mini Kit (Qiagen, Hilden, Germany), following the manufacturer’s recommendations. Each species was amplified from a single voucher specimen, but a second voucher was also included for some taxa. The three cpDNA regions were amplified using polymerase chain reaction (PCR) and sequenced for each taxon. The complete *ndh*F gene, coding NADH dehydrogenase subunit F, was amplified with a battery of primers in different combinations in four overlapping fragments using the primer pairs specified by [7,31]: 5F–536R, 536F–972R, 972F–1666R, and 1666F–3R; the *rpl*16 region, corresponding to the intron and partial sequences of the gene encoding ribosomal protein L16 [32,33,34], was amplified in two fragments using primers F71 [35] and R1661 [32], combined with the internal ones F584 and R584 [36]; finally, the *trn*L intron and *trn*L-F spacer were amplified by PCR in two fragments using the primers C, D, E, and F of [37].

PCR reactions were performed in a 25 µL final volume with 50–100 ng of template DNA, 5 µL of Green Promega GoTaq^®^ buffer (5 u/µL), 0.5 µL of MgCl_2_ (25 mM), 1.25 µL of dNTP (10 mM), 1 µL of each primer (10 pM), and 0.3 µL of Taq polymerase (5 u/µL) provided by Promega (Madison, Wisconsin, U.S.A.). For the species that failed this protocol, variations in MgCl_2_ (0.5–1 µL) and total DNA dilutions (1:5, 1:10 and 1:50) were used. The reactions were carried out using the following parameters: one cycle of 95 °C for 2 min, 39 cycles of 95 °C for 30 s, 48 °C for 30 s, and 72 °C for 1.5 min, and a final extension cycle of 72 °C for 10 min. The PCR products were run out on a 1% TBE agarose gel stained with SYBR Safe DNA gel stain (Invitrogen) and visualized in a blue-light transilluminator. The PCR products were purified, and automated sequencing was performed by Macrogen, Inc. (Seoul, South Korea). Forward and reverse strands were sequenced for all fragments, with a minimum overlap of 80%.

#### 4.1.3. Phylogenetic Analyses

The sequences were edited and assembled in MEGA v. 7.0 [38]. To check the accuracy, all sequences were translated to aminoacids and the point substitutions were checked against the original sequencing trace file. Alignments were generated using Clustal X v. 2 [39] under the default settings and, when necessary, manually improved. The phylogenetic reconstruction was based on parsimony (MP) [40], maximum likelihood (ML) [41,42], and Bayesian inference (BI) [43] methods. In all analyses, gaps were considered as missing data. The best-fitting nucleotide substitution models for the three regions were determined by using the Akaike information criterion (AIC), as implemented in jModeltest 2.1.1 [44]: TVM+I+G (*ndh*F) and TPM1uf+I+G (*rpl*16 and *trn*L-F).

For the three approaches, each cpDNA region matrix was analyzed separately before the analysis of the combined datasets. Separate and combined parsimony analyses were conducted in TNT ver. 1.1 [45]. All characters were equally weighted and treated as unordered. A heuristic search was conducted with 1000 random addition sequences, tree bisection and reconnection (TBR) branch swapping, saving up to 15 trees per replicate. The resulting trees were then submitted to a second-round search of TBR branch swapping to completion. Nonparametric bootstrap support (BS) [46] was estimated using 10,000 pseudo-replicates, and the same parameters were used in our MP analyses.

The ML analyses were performed in RAxML-HPC2 on XSEDE (v. 8.2.12) [47] through the Cyberinfrastructure for Phylogenetic Research (CIPRES) Portal v. 3.3 [48]. For these analyses, we used non-parametric bootstrap (BS) analysis and searched for the best-scoring ML tree in a single run [49]. To this end, we performed 1000 rapid bootstrap inferences with a subsequent search of the maximum likelihood tree, using the GTRGAMMA nucleotide substitution model [49], individual per-site substitution rates (-c), and default setting of likelihood acceptance (-e), 25 and 0.1, respectively. Individual and combined BI analyses were conducted in MrBayes v. 3.2.7a [50] through the CIPRES Portal [48] with nst = 6 and rates= invgamma, unlinking models across loci for combined analyses. The datasets were analyzed in two parallel runs of four simultaneous Markov chains for 10 million generations, sampling every 1000 generations and using the default parameters. Convergence and effective sample size (ESS) of the runs were assessed by checking the parameters in Tracer v.1.7 [51]. After discarding the initial 2500 trees of each run as burn-in (25%), the remaining trees (15,002) were used to generate a 50% majority-rule consensus tree. The cutoff for strong support in the Bayesian analyses was 0.95 (roughly equal to *p* < 0.05), and posterior probabilities and values below 0.8 were considered not supported.

### 4.2. Anatomical and Morphological Analyses

The anatomical and taxonomic studies were based on bibliographical research including recent studies (e.g., [52,53], among others) and original descriptions, as well as analyses of herbarium specimens housed in B, BR, C, FT, G, K, MO, NY, P, UBT, US, and W [54]. We examined types in person and the images are available online at the JSTOR Global Pants website (http://plants.jstor.org, accessed on 10 October 2022) and/or at the websites of aforementioned herbaria. The protologues of all taxa were checked. Unless otherwise stated, the specimen designated as the lectotype was that which matches the protologue, corresponds to the current usage of the plant name, and is in the best preservation condition, according to the modern rules of the International Code of Nomenclature (ICN) [55]. For each name, the place of valid publication is given followed by the holotype or lectotype and an explanation of the nomenclatural decisions made.

The spikelets and upper anthecia were viewed using a Philips XL 30 TMP at an accelerating voltage of 80 kV in the scanning electron microscope. The foliar anatomy was analyzed in the second leaf below the inflorescence. The histological preparations were made from the herbarium materials that were rehydrated by submerging them in water with a commercial detergent and heating them in an oven at 50 °C for 24 h, followed by fixing them in FAA. The cross-sectional leaf anatomy was determined from the hand-sectioned leaf blades, after clarifying with 50% sodium hypochlorite, being stained with Safranin and Alcian Blue [56], and being mounted in glycerin jelly. The observations and measurements were made using a Wild M20 optical light microscope and photomicrographs were taken using Axio Vs40 V 4.8.2.0 (Carl Zeiss). The terminology for the anatomical characters and descriptions were based on [57,58].

## 5. Conclusions

Our study allowed us to exclude three species of the Melinidineae from the genus *Panicum* on the basis of morphological and molecular characters, establishing a new genus in the subtribe and transferring the remaining two species to the genus *Megathyrsus*. In addition, we present here a summary of the main features of the genera of the subtribe, including a key to distinguishing the genera of the Melinidineae.

## Data Availability

All aligned data matrices and trees of the separate and combined datasets from the three methods of analysis are available at the Repositorio Institucional CONICET Digital under the following link: http://hdl.handle.net/11336/17622, accessed on 23 December 2022.

## Supplementary Materials

The following supporting information can be downloaded at https://www.mdpi.com/article/10.3390/plants12020399/s1, Table S1: Descriptive statistics for each separate data partition and combined matrices used in the parsimony analyses. Table S2: Taxa, voucher information, and GenBank accession numbers for *ndh*F, *rpl*16, and *trn*L-F, respectively.
