# Supplementary material for: Systematics and Phylogenetic Placement of Panicum L. Species within the Melinidinae Based on Morphological, Anatomical, and Molecular Data (Poaceae, Panicoideae, Paniceae)
Source: Plants (Basel). 2023 Jan 14;12(2):399. doi: 10.3390/plants12020399 (PMC10375907; doi:10.3390/plants12020399)
Supplement: Supplementary file 1 [file plants-12-00399-s001.zip › plants-2119059 supplement final.pdf]

Table S1. Descriptive statistics for each separate data partition and combined matrices used in parsimony analyses.

DNA region / dataset	*ndhF*	*rpl16*	*trn*L-F	Total dataset
Melinidinae (number of taxa)	111	74	72	111
Outgroup (number of taxa)	14	9	11	14
Alignment length (bp)	2130	1187	1172	4490
Parsimony informative sites	297 (13.94%)	115 (9.68%)	127 (10.83%)	539 (12%)
Tree length	661	221	247	1174
Consistency index (CI)	0.561	0.665	0.692	0.587
Retention index (CR)	0.856	0.793	0.846	0.831

Table S2. Taxa, voucher information, and GenBank accession numbers for *ndh*F, *rpl*16, and *trn*L-F, respectively. Species/sequences not analyzed in [11] are indicated with an asterisk (*); sequences obtained for this study are indicated with double asterisk (**) and regions with no sequence data are indicated with a dash (–); herbarium acronyms follow [54].

Ingroup (Melinidinae)	*ndh*F	*rpl*16	*trn*L-F
Brachiaria ambigens Chiov.*	MF998656*	–	–
Brachiaria humbertiana A. Camus (1)*	LN908062*	–	–
Brachiaria humbertiana (2)*	MF998517*	–	–
Brachiaria nana Stapf*	MF998514*	–	–
Brachiaria pseudodichotoma Bosser (1)	GU594627	GU594521	GU594587
Brachiaria pseudodichotoma (2)	MF998520*	–	–
Brachiaria subrostrata A. Camus	MF998492*	–	–
Brachiaria umbellata (Trin.) Clayton	MF998493*	–	–
Chaetium bromoides (J. Presl) Benth.	AY029626	GU594519	AF509962
Eriochloa acuminata (J.Presl) Kunth	GU594634	GU594528	GU594594
Eriochloa distachya Kunth	GU594617	GU594510	GU594573
Eriochloa fatmensis (Hochst. & Steud.) Clayton*	MF998531*	HQ999912*	–
Eriochloa meyeriana (Nees) Pilg.	GU594623	GU594516	GU594583
Eriochloa montevidensis Griseb.	GU594598	GU594491	GU594535
Eriochloa nana Arriaga	GU594605	GU594498	GU594553
Eriochloa polystachya Kunth	GU594629	GU594523	GU594589
Eriochloa procera (Retz.) C.E. Hubb. *	MF998529*	–	–
Eriochloa punctata (L.) Ham.	FJ486528	FJ486555	GU594554

Eriochloa sericea (Scheele) Munro ex Vasey	GU594635	GU594529	GU594595
Eriochloa setosa (A. Rich.) Hitchc.	GU594625	GU594518	GU594585
Eriochloa subulifera Stapf*	MF998530*	–	–
Eriochloa villosa (Thunb.) Kunth*	KM538704*	–	KX372423*
Leucophrys mesocoma (Nees) Rendle	GU594628	GU594522	GU594588
Megathyrsus infestus (Andersson) B.K. Simon & S.W.L. Jacobs	GU594618	GU594511	GU594575
Megathyrsus maximus (Jacq.) B.K. Simon & S.W.L. Jacobs	LN908130*	FJ486550	GU594547
Melinis minutiflora P. Beauv. (1)	FJ486524	FJ486551	GU594549
Melinis minutiflora (2)	AM849212*	–	–
Melinis minutiflora (3)	MF998508*	–	–
Melinis repens (Willd.) Zizka (1)	FJ486532	FJ486559	GU594561
Melinis repens (2)	KP878958*	KP879020*	MT263048*
Moorochloa eruciformis (Sm.) Veldkamp	FJ486525	FJ486552	GU594550
Moorochloa malacodes (Mez & K.Schum.) Veldkamp	FJ486536	FJ486564	GU594576
Panicum deustum Thunb. (1)<br>= *Batochloa deusta* (Thunb.) Salariato & Zuloaga	GU594631	GU594525	GU594591
Panicum deustum (2)<br>Ethiopia, Gamu-Gofa region, *I. Friis 9510* (K)	OQ383273**	OQ383280**	OQ383277**
Panicum deustum (3)<br>Democratic Republic of the Congo, Beni, *J. Bequaert 3398* (BR)	OQ383274**	–	OQ383278**
Panicum trichocladum Hack. ex K.Schum. (1)*<br>= *Megathyrsus trichocladum* (Hack. ex K. Schum.) Salariato & Zuloaga	LN908153*	–	–
Panicum trichocladum (2)<br>Democratic Republic of the Congo, Burundi, *J. Lambinon 7573* (BR)	OQ383275**	OQ383281**	–
Panicum vollesenii Renvoize*<br>= *Megathyrsus vollesenii (*Renvoize) Salariato & Zuloaga<br>Tanzania, Liwale District, *K. Vollesen 3871* (K)	OQ383276**	OQ383282**	OQ383279**
Rupichloa acuminata (Renvoize) Salariato & Morrone	FJ486540	FJ486568	GU594562
Rupichloa decidua (Morrone & Zuloaga) Salariato & Morrone (1)	FJ486526	FJ486553	GU594551

Rupichloa decidua (2)	FJ486539	FJ486567	–
Scutachne dura (Griseb.) Hitchc. & Chase	GU594616	GU594509	GU594572
Thuarea involuta (G. Forst.) R. Br. ex Sm. (1)	GU594624	GU594517	GU594584
Thuarea involuta (2)	MF998491*	–	–
Thuarea perrieri A. Camus*	MF998490*	–	–
Tricholaena monachne (Trin.) Stapf & C.E. Hubb. (1)	FJ486535	FJ486563	GU594574
Tricholaena monachne (2)	HE577883*	–	–
Tricholaena monachne (3)	MF998509*	–	–
Urochloa adspersa (Trin.) R.D. Webster	GU594599	GU594492	GU594537
Urochloa arizonica (Scribn. & Merr.) Morrone & Zuloaga	GU594614	GU594507	GU594569
Urochloa arrecta (Hack.) Morrone & Zuloaga (1)	FJ486517	FJ486544	GU594536
Urochloa arrecta (2)	MF998513*	–	MK424972*
Urochloa arrecta (3)	MF998523*	–	MK424973*
Urochloa bovonei (Chiov.) A.M. Torres & C.M. Morton	FJ486530	FJ486557	GU594557
Urochloa brachyura (Hack.) Stapf*	MF998533*	HQ999943*	–
Urochloa brizantha (A. Rich.) R.D. Webster (1)	FJ486520	FJ486546	GU594542
Urochloa brizantha (2)	LN908057*	–	–
Urochloa brizantha (3)	MF998528*	–	–
Urochloa comata (Hochst. ex A. Rich.) Sosef	GU594620	GU594513	GU594580
Urochloa decumbens (Stapf) R.D. Webster (1)	FJ486529	FJ486556	GU594555
Urochloa decumbens (2)	MF998527*	–	–
Urochloa deflexa (Schumach.) H. Scholz (1)	FJ486534	FJ486562	GU594571
Urochloa deflexa (2)	MF998524*	–	–
Urochloa dictyoneura (Fig. & De Not.) Veldkamp	FJ486523	FJ486549	GU594546
Urochloa discifera (E. Fourn.) Morrone & Zuloaga	GU594604	GU594497	GU594548
Urochloa distachya (L.) T.Q. Nguyen (1)	FJ486518	FJ486545	GU594538
Urochloa distachya (2)	LN908059*	–	–
Urochloa dura (Stapf) A.M. Torres & C.M. Morton	GU594610	GU594503	GU594564
Urochloa echinolaenoides Stapf	GU59463	GU594526	GU594592

Urochloa foliosa (R. Br.) R.D. Webster	GU594619	GU594512	GU594578
Urochloa fusca (Sw.) B.F. Hansen & Wunderlin	GU594601	GU594494	GU594540
Urochloa glumaris (Trin.) Veldkamp (1)	GU594626	GU594520	GU594586
Urochloa glumaris (2)	MF998518*	–	–
Urochloa holosericea (R. Br.) R.D. Webster	GU594630	GU594524	GU594590
Urochloa humidicola (Rendle) Morrone & Zuloaga (1)	FJ486521	FJ486547	GU594544
Urochloa humidicola (2)	MF998526*	–	–
Urochloa jubata (Fig. & De Not.) Sosef	FJ486531	FJ486558	GU594559
Urochloa lachnantha (Hochst.) A.M. Torres & C.M. Morton	GU594615	GU594508	GU594570
Urochloa lata (Schumach.) C.E. Hubb.	FJ486533	FJ486561	GU594567
Urochloa leersioides (Hochst.) A.M. Torres & C.M. Morton (1)	GU594608	GU594501	GU594560
Urochloa leersioides (2)	MF998516*	–	–
Urochloa lorentziana (Mez) Morrone & Zuloaga	FJ486522	FJ486548	GU594545
Urochloa meziana (Hitchc.) Morrone & Zuloaga	GU594602	GU594495	GU594541
Urochloa mollis (Sw.) Morrone & Zuloaga	GU594597	GU594490	GU594534
Urochloa mosambicensis (Hack.) Dandy (1)	FJ486516	FJ486542	GU594532
Urochloa mosambicensis (2)	MF998522*	–	–
Urochloa mutica (Forssk.) T.Q. Nguyen	MF998515*	GU594530	GU594596
Urochloa nigropedata (Munro ex Ficalho & Hiern) A.M. Torres & C.M. Morton	GU594606	GU594499	GU594556
Urochloa oblita (Swallen) Morrone & Zuloaga	FJ486538	FJ486566	GU594579
Urochloa panicoides P. Beauv. (1)	FJ486519	EU920055	EU920056
Urochloa panicoides (2)	MF998519*	–	–
Urochloa paucispicata (Morong) Morrone & Zuloaga	FJ486527	FJ486554	GU594552
Urochloa plantaginea (Link) R.D. Webster	MF998511*	FJ486543	GU594533
Urochloa platynota (K. Schum.) Pilg.	GU594609	GU594502	GU594563
Urochloa platyphylla (Munro ex C. Wright) R.D. Webster	GU594600	GU594493	GU594539
Urochloa ramosa (L.) T.Q. Nguyen*	MF998534*	–	–
Urochloa reptans (L.) Stapf	LN908063*	–	–

Urochloa rudis Stapf*	GU594633	GU594527	GU594593
Urochloa ruziziensis (R. Germ. & C.M. Evrard) Crins	GU594603	GU594496	GU594543
Urochloa semiundulata (Hochst. ex A. Rich.) Ashal. & V.J. Nair	LN908064*	HQ999876*	–
Urochloa serrata (Thunb.) Sosef (1)	GU594607	GU594500	GU594558
Urochloa serrata (2)	MF998532*	–	–
Urochloa setigera (Retz.) Stapf	GU594613	GU594506	GU594568
Urochloa subquadripara (Trin.) R.D. Webster (1)	GU594612	GU594505	GU594566
Urochloa subquadripara (2)	MF998512*	–	–
Urochloa subulifolia (Mez) Torres Gonz. & C.M. Morton	GU594611	GU594504	GU594565
Urochloa texana (Buckley) R.D. Webster	FJ486537	FJ486565	GU594577
Urochloa xantholeuca (Hack.) H. Scholz*	MF998655*	HQ999877*	–
Yvesia madagascariensis A. Camus (1)	MF998494*	–	–
Yvesia madagascariensis (2)	GU594636	–	–
**Outgroup**	***ndh*F**	***rpl*16**	***trn*L-F**
Aakia tuerckheimii (Hack.) J.R. Grande	KF982003	–	–
Anthaenantiopsis rojasiana Parodi	MT180181	KF852999	MT180272
Cenchrus ciliaris L.	AY029625	HQ999878	EU940006
Cenchrus echinatus L.	AF499151	EU931890	EU940007
Cenchrus setaceus (Forssk.) Morrone	AY029673	–	EU940000
Panicum antidotale Retz.	AY188456	–	–
Panicum ligulare Nees ex Trin.	MG581772	–	–
Panicum miliaceum L.	AY188472	–	JQ972957
Panicum repens L.	HE575806	EU920053	GU594531
Panicum venosum Swallen	GU594622	GU594515	GU594582
Paspalum notatum Flüggé	KF852941	KF853041	MT180275
Setaria geminata (Forssk.) Veldkamp	AY029662	EU931861	EU939981
Setaria lachnea (Nees) Kunth	EU741952	EU931863	EU939984
Setaria viridis (L.) P. Beauv.	U21976	–	AF499156
